# Acute Effects of a 30 s Maximal Anaerobic Cycling Test on Change-of-Direction Performance During Recovery

**DOI:** 10.3390/life16010072

**Published:** 2026-01-03

**Authors:** Yeliz Pehlivan, Nedim Malkoç, Erdal Bal, Hamza Küçük

**Affiliations:** 1 Department of Exercise and Sport Sciences, Hamidiye Faculty of Life Sciences, Istanbul Health Sciences University, Istanbul 34668, Türkiye; yeliz.yol@sbu.edu.tr (Y.P.); nedim.malkoc@sbu.edu.tr (N.M.); erdal.bal@sbu.edu.tr (E.B.); 2 Yasar Dogu Faculty of Sport Sciences, Ondokuz Mayis University, Samsun 55200, Türkiye

**Keywords:** maximal anaerobic power, recovery, sex differences, change-of-direction

## Abstract

This study aimed to examine the acute effects of a 30 s maximal anaerobic cycling test on change-of-direction performance during recovery and to determine whether these effects differed between genders and groups. A total of 64 university students who were actively participating in sports participated in the study (33 males, age: 21.0 ± 1.03 years; 31 females, age: 20.38 ± 0.91 years). Participants were assigned to an experimental or control group. Planned change-of-direction (COD) performance was assessed using the Illinois test at baseline and at the 4th, 6th, 8th, and 10th minutes of recovery with a photocell timing system. Data were analyzed using a mixed-design ANOVA with Time as a within-subject factor and Group and Sex as between-subject factors. Significant main effects of Time, Group, and Sex were observed (*p* < 0.05), indicating time-dependent changes in COD performance, overall performance differences between groups, and sex-related differences in Illinois test times. However, no significant Time × Group, Time × Sex, or Time × Group × Sex interactions were found (*p* > 0.05), suggesting that the pattern of recovery over time was similar across groups and sexes. These findings indicate that a 30 s maximal anaerobic cycling test induces acute fatigue that affects planned change-of-direction performance and that recovery of Illinois test performance is not completed within 10 min, with similar recovery patterns observed across groups and sexes.

## 1. Introduction

Agility is the integration of individuals’ physical abilities (acceleration, deceleration, strength, coordination) with their cognitive skills (perception, decision-making, responding to unexpected stimuli) [1,2,3,4,5]. Advanced agility performance enables athletes to quickly adapt to their opponents’ movements and environmental changes, which is crucial for both offensive and defensive maneuvers [4,5,6]. Conversely, low agility performance is associated with a higher risk of injury in both individual and team athletes, making agility development important for both performance and injury prevention [7,8]. Despite the frequent interchangeable use of the terms agility and change-of-direction (COD) in the literature, these constructs represent distinct performance qualities [9,10,11,12,13]. Planned COD performance refers to the ability to rapidly decelerate and re-accelerate in a known movement pattern without a reactive or perceptual component. The Illinois test is widely used as a field-based assessment of planned COD performance due to its high reliability and practical applicability [12,14,15]. The test involves repeated accelerations, decelerations, and 180° turns within a predetermined course, providing information about an athlete’s capacity to perform rapid directional changes under controlled conditions. However, because the movement pattern is known in advance, the Illinois test assesses planned COD performance rather than reactive agility or perceptual–cognitive components [9,10,11,12,13].

Various protocols are used to evaluate fatigue and anaerobic capacity in athletes, among maximal anaerobic power tests being the most commonly applied. These tests assess both alactic and lactic anaerobic energy system contributions and are typically performed using short-duration all-out efforts on cycle ergometers [16,17]. Such protocols impose substantial metabolic and neuromuscular stress, resulting in acute fatigue that may transiently impair subsequent motor performance.

The literature has extensively investigated the chronic effects of anaerobic exercise protocols in terms of physiological responses, perceived recovery, and performance outcomes. Research has primarily focused on relationships between power output, sprint performance, repeated sprint ability, aerobic and anaerobic adaptations, and metabolic responses following high-intensity or maximal exercise. Although several studies have reported that COD performance may be impaired following acute anaerobic or sprint-based activities, most have examined recovery over very short time intervals or during repeated sprint protocols. Consequently, there remains limited evidence regarding the acute recovery profile of planned COD performance following a single 30 s maximal anaerobic cycling test, particularly across multiple early recovery time points beyond the first few minutes and with consideration of sex and group allocation [13,18,19,20,21,22,23,24].

Therefore, the aim of this study was to examine changes in planned change-of-direction performance at different recovery time points (4th, 6th, 8th, and 10th minutes) following a 30 s maximal anaerobic cycling test and to determine whether these responses differed according to sex and group allocation. Understanding the acute fatigue and recovery responses of COD performance may provide practical insights for training load management, recovery scheduling, and performance monitoring in exercise and sport science contexts.

## 2. Materials and Methods

### 2.1. Research Group

This study was conducted on 31 females and 33 males aged 18–25 who had been engaging in regular physical activity for at least 1 year at the University of Health Sciences.

### 2.2. Personal Information Form

The personal information form was created by the researcher. In this information form, questions such as the participants’ age, body weight, and whether they had chronic diseases were asked according to the purpose of the study, and a document was signed that they voluntarily participated. The research was approved by the Scientific Research Ethics Committee that the research structure was in accordance with the Helsinki Declaration on ‘Ethical Principles in Medical Research on Humans’ (2025/12-12/20).

### 2.3. Research Design

This study employed an acute, experimental, quantitative design. Participants attended the laboratory on two occasions. The first visit consisted of a familiarization session, during which participants practiced the Illinois test and the cycling protocol to minimize potential learning effects. No data were recorded during this session. One week later, participants returned to the laboratory for the experimental session. After a standardized 10 min active warm-up, participants were randomly assigned to either an experimental group or a control group. Randomization was performed using a simple random allocation method. One week later, participants returned to the laboratory for the experimental session. After a standardized 10 min active warm-up, participants were randomly assigned to either an experimental group or a control group. Randomization was performed using a simple random allocation method. The experimental group completed a 30 s all-out maximal anaerobic cycling test on a Wattbike cycle ergometer (Wattbike Pro/Trainer, Nottinghamshire (England). Following the 30 s all-out maximal anaerobic cycling test, participants remained in passive recovery for 2 min. Planned change-of-direction performance was subsequently assessed at the 4th, 6th, 8th, and 10th minutes of recovery. The control group performed the same standardized warm-up protocol but did not complete the anaerobic cycling test. After the warm-up, participants rested passively for two minutes and then performed the Illinois test at the same time points (4th, 6th, 8th, and 10th minutes) as the experimental group.

**Figure 1:**
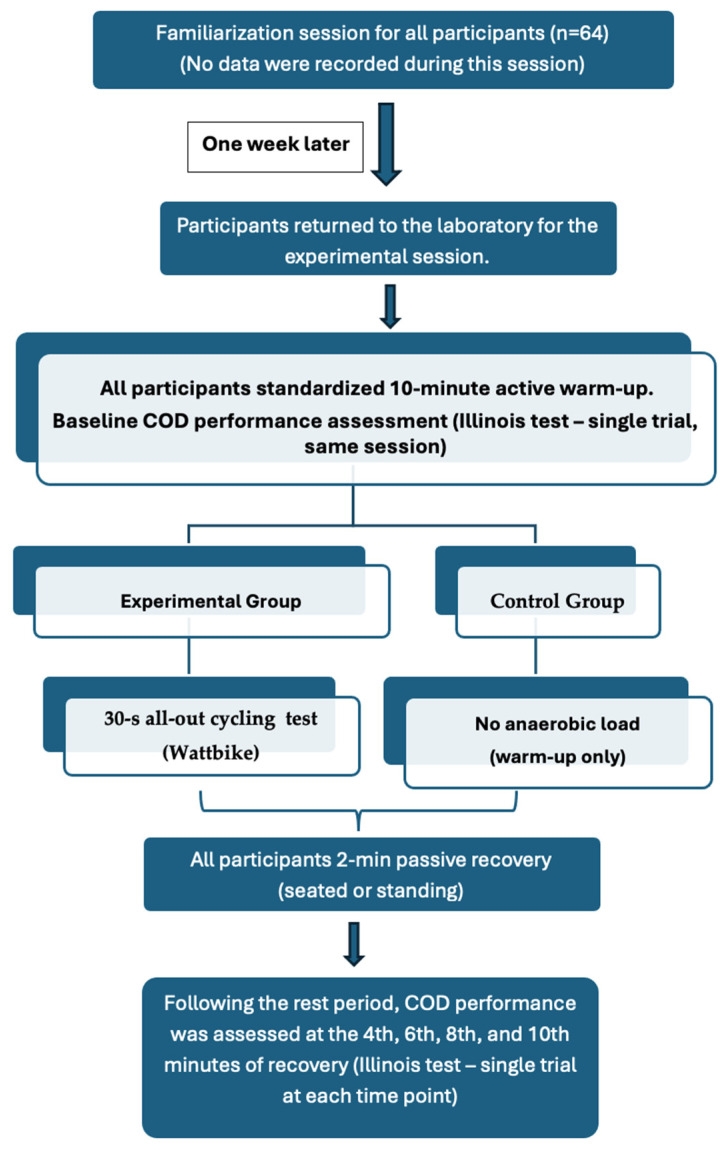
Flowchart of the experimental procedure illustrating the familiarization session, baseline assessment, group allocation, anaerobic cycling protocol, passive recovery period, and timing of planned change-of-direction (COD) performance measurements.

A schematic overview of the experimental procedure, including the familiarization session, baseline assessment, group allocation, anaerobic cycling protocol, passive recovery period, and the timing of planned change-of-direction (COD) performance measurements, is presented in Figure 1. This flowchart summarizes the sequence and timing of all experimental steps and clarifies that baseline and recovery measurements were obtained within the same experimental session.

### 2.4. Data Collection Tools

#### 2.4.1. Body Weight and Height Measurement

Height and body weight were measured using a digital scale (Seca 769, Hamburg, Germany). Participants were asked to wear thin leggings and a t-shirt. Body weight was recorded in kg and height in cm.

#### 2.4.2. Body Mass Index (BMI)

BMI was determined by dividing an individual’s body weight in kilograms by the square of their height in meters, using the standard formula: BMI = weight (kg)/height^2^ (m).

#### 2.4.3. 30 s Anaerobic Power Test

The 30 s sprint test was conducted using a Wattbike Pro cycle ergometer (Wattbike Ltd., Nottingham, UK). It was employed as a fatigue-inducing protocol rather than a performance assessment.

Warm-Up: Prior to the cycling test, participants completed a standardized 10 min active warm-up, which consisted of low-to-moderate intensity cycling and dynamic movements, as described in the Research Design section. Following the warm-up, participants rested passively for 2 min before initiating the anaerobic cycling test.

Test Protocol: The test was performed from a stationary start position. Participants were instructed to pedal maximally for 30 s, applying an all-out effort throughout the entire duration of the test. Strong verbal encouragement was provided to ensure maximal effort was maintained during the sprint. The air resistance (air-brake) setting of the Wattbike Pro was individually adjusted based on body mass in accordance with previously described Wattbike sprint testing procedures [25]. Resistance settings were kept constant throughout the test, and all participants used the same ergometer to ensure consistency across trials. This protocol was selected due to its ability to impose a substantial metabolic and neuromuscular load, leading to acute fatigue, which allowed examination of subsequent changes in planned change-of-direction performance during the recovery period.

All participants were instructed to maintain their regular training routines up to 48 h prior to each test session. Additionally, they were asked to avoid intense physical activity within the 24 h before testing and to abstain from caffeine for at least 12 h. All assessments were conducted on the same cycle ergometer and scheduled at consistent times of day (±1 h) to reduce potential effects of circadian fluctuations. Participants were required to arrive well-rested and properly hydrated.

#### 2.4.4. Illinois Change-of-Direction Test

This test involves a course that measures 10 m in length and 5 m in width, with four cones placed in a straight line at 3.3 m intervals in the center of the course. The test consists of a total of 40 m of linear sprinting and approximately 20 m of slalom running with 180-degree turns around the cones. A dual-gate photoelectric timing system with 0.01 s precision was positioned at the start and finish lines (Queensland, Australia). Once participants assumed a ready position, they began the test from the designated start point, and the time taken to complete the course was recorded in seconds. The test was repeated at predetermined time intervals [24].

### 2.5. Data Analysis

All statistical analyses were performed using SPSS version 27.0 (IBM Corp., Armonk, NY, USA). Data are presented as mean ± standard deviation (SD). The normality of data distribution was assessed using the Shapiro–Wilk test, and supported by examination of skewness and kurtosis values, which were considered acceptable within the ±1 range [26]. Changes in planned change-of-direction (COD) performance across time were analyzed using a mixed-design analysis of variance (ANOVA), with Time (baseline, 4th, 6th, 8th, and 10th minutes) as the within-subject factor and Group (experimental vs. control) and Sex (female vs. male) as between-subject factors. The assumption of sphericity was evaluated using Mauchly’s test, and when violated, the Greenhouse–Geisser correction was applied. When significant main effects were detected, Bonferroni-adjusted post hoc comparisons were performed. Effect sizes were calculated using partial eta squared (η^2^*p*) and interpreted as small (≥0.01), medium (≥0.06), or large (≥0.14). The level of statistical significance was set at *p* < 0.05. In the absence of significant interaction effects, results were interpreted based on main effects only, and no differential recovery patterns between groups or sexes were inferred. An a priori power analysis was conducted using G*Power (3.1.9.4) software based on an expected medium effect size (Cohen’s d = 0.50), an alpha level of 0.05, and a power of 0.85, indicating that a sample size of approximately 31 participants per group was sufficient to detect meaningful differences.

## 3. Results

The descriptive statistics of the participants are presented in Table 1.

Descriptive statistics for anthropometric characteristics are presented in Table 1. The study included 64 participants (48.44% female and 51.56% male). Mean height, body mass, and BMI were 172.88 ± 9.48 cm, 66.49 ± 12.55 kg, and 22.09 ± 2.74 kg/m^2^, respectively. The performance values across the five time points are presented in Table 2.

Descriptive statistics for planned change-of-direction (COD) performance assessed by the Illinois test across baseline and recovery time points are shown in Table 2. In the experimental group, Illinois test times increased at all post-exercise time points compared with baseline in both female and male participants, indicating a reduction in COD performance following the maximal anaerobic cycling test. In the control group, Illinois test times remained relatively stable across time points.

The results of the mixed-design ANOVA examining the effects of Time, Group, and Sex on Illinois test performance are presented in Table 3. A significant main effect of Time was observed (F (4,58) = 6.48, *p* < 0.001, η^2^*p* = 0.309), indicating time-dependent changes in COD performance. Significant main effects of Group (F (1.61) = 7.15, *p* = 0.010, η^2^*p* = 0.105) and Sex (F (1.61) = 14.47, *p* < 0.001, η^2^*p* = 0.192) were also found, with overall differences in Illinois test times between experimental and control groups and between female and male participants. The results of the mixed-design ANOVA examining the effects of Time, Group, and Sex on Illinois test performance are presented in Table 3. A significant main effect of Time was observed (F (4.58) = 6.48, *p* < 0.001, η^2^*p* = 0.309), indicating time-dependent changes in COD performance. Significant main effects of Group (F (1.61) = 7.15, *p* = 0.010, η^2^*p* = 0.105) and Sex (F (1.61) = 14.47, *p* < 0.001, η^2^*p* = 0.192) were also found, with overall differences in Illinois test times between experimental and control groups and between female and male participants.

**Figure 2:**
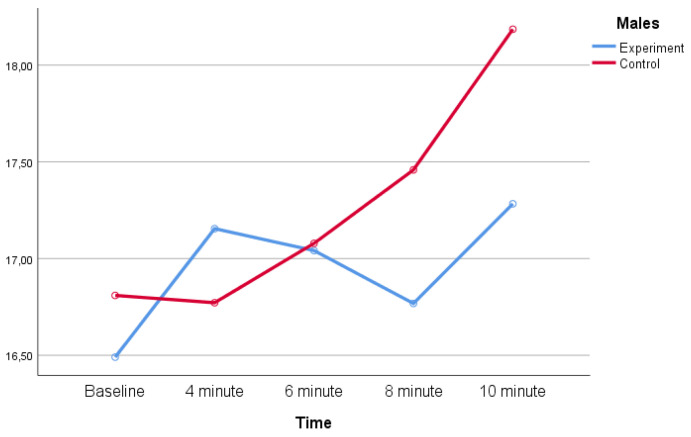
Planned change-of-direction (COD) performance (Illinois test time, s) of male participants in the experimental and control groups across measurement time points.

**Figure 3:**
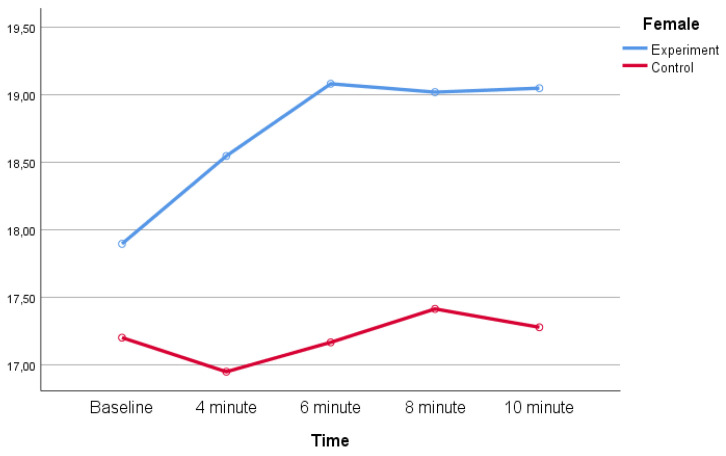
Planned change-of-direction (COD) performance (Illinois test time, s) of female participants in the experimental and control groups across measurement time points.

The distribution of mean Illinois test times across groups and sexes at each measurement point is illustrated in Figure 2 and Figure 3.

## 4. Discussion

The aim of this study was to examine the acute effects of a 30 s maximal anaerobic cycling test on planned change-of-direction (COD) performance during the early recovery period and to determine whether these responses differed according to sex or group allocation. The main finding of the study was that planned COD performance, assessed using the Illinois test, was temporarily impaired following the anaerobic protocol, with increased test durations at multiple recovery time points. This finding suggests that short-duration maximal anaerobic cycling exercise causes acute fatigue sufficient to negatively impact subsequent COD performance.

Jakobsson et al. [22] reported that participants were unable to recreate change-of-direction performance immediately following cycling fatigue, but that COD performance was recovered after a two-minute passive recovery period following fatigue. Consistent with these findings, the present study demonstrated a transient impairment in planned change-of-direction performance during the early recovery phase following a 30 s maximal anaerobic cycling test and expanded on the existing literature by examining change-of-direction performance at multiple early recovery time points (4th, 6th, 8th, and 10th minutes).

The acute reduction in COD performance observed in the present study may be associated with fatigue-induced alterations in neuromuscular function. Planned COD performance relies heavily on eccentric muscle actions and rapid deceleration–reacceleration phases, which are strongly dependent on effective utilization of the stretch–shortening cycle (SSC). Acute fatigue may temporarily disrupt SSC efficiency, leading to impaired braking and acceleration mechanics. Previous evidence suggests a close relationship between power production capacity and eccentric hamstring strength, indicating that fatigue-related impairments in eccentric muscle function may negatively affect COD performance [27,28,29].

Supporting this interpretation, Cortes et al. [29] reported that fatigued athletes exhibited reduced knee flexion and increased hip flexion during directional changes, biomechanical alterations that may limit effective SSC utilization. Such movement strategy changes could contribute to the observed decrements in COD performance during the early recovery period following maximal anaerobic exercise. Although the present study did not directly assess biomechanical or neuromuscular variables, the time-dependent impairment in COD performance is consistent with these previously reported fatigue-related mechanisms.

The findings of the present study can also be interpreted within the broader framework of neuromuscular adaptations known to underpin change-of-direction (COD) performance. Chronic training studies have consistently demonstrated that improvements in COD ability are primarily driven by neuromuscular and mechanical adaptations, including enhanced motor unit recruitment, increased firing frequency, and improved efficiency of the stretch–shortening cycle (SSC). For instance, Kargarfard et al. [30] reported that a combined plyometric and speed training program elicited significant improvements in COD performance in young soccer players, highlighting the critical role of explosive force production and neuromuscular coordination during directional changes. Although the findings of Kargarfard et al. [30] reflect chronic training-induced adaptations rather than acute responses, these underlying mechanisms may provide a relevant physiological framework for interpreting the acute, fatigue-related changes observed in the present study. Specifically, the transient impairment in COD performance following maximal anaerobic cycling may be attributed to temporary disruptions in neuromuscular coordination, which appear to be progressively restored as recovery advances.

Another finding of the study is the absence of a significant difference in Time × Group or Time × Gender interactions; this indicates that the temporal pattern of change in COD performance during recovery is similar between the experimental and control groups and between female and male participants. This suggests that although overall performance levels differ between groups and genders, the dynamics of acute recovery following the fatigue-inducing protocol are comparable. Therefore, we can say that acute fatigue-related impairments in planned COD performance may exhibit similar temporal characteristics regardless of gender. Regarding Group and Gender, significant main effects were found; this indicates overall differences in COD performance levels between the experimental and control groups and between female and male participants. These may be related to differences in baseline performance, anthropometric characteristics, or movement strategies rather than different responses to the acute anaerobic cycling protocol. Additionally, a significant Group × Gender interaction shows that the magnitude of gender-dependent performance differences varies between groups; however, since this interaction is not time-dependent, we can interpret it as a difference in overall performance levels rather than a difference in recovery patterns. In contrast to these study results, a study investigating the effect of a 4 s sprint protocol applied in 4 sets on a bicycle ergometer on COD (*t*-test protocol) found no significant difference between genders [26].

From a practical perspective, these findings highlight that planned COD performance may be temporarily impaired following short-duration maximal anaerobic exercise. Coaches and practitioners should therefore consider the potential negative impact of acute fatigue on COD tasks when designing training sessions or scheduling performance assessments. Ensuring adequate recovery following maximal anaerobic efforts may be essential for optimizing the execution of planned change-of-direction movements.

## 5. Conclusions

This study demonstrated that a 30 s maximal anaerobic cycling test induces a transient impairment in planned change-of-direction (COD) performance during the early recovery period, as reflected by increased Illinois test times at multiple post-exercise time points. These findings indicate that short-duration all-out anaerobic exercise imposes sufficient acute fatigue to negatively affect subsequent COD performance. Although overall COD performance levels differed between experimental and control groups and between female and male participants, the absence of significant time-dependent interaction effects suggests that the temporal pattern of recovery was similar across groups and sexes. Collectively, these results highlight that acute fatigue-related changes in planned COD performance follow comparable recovery dynamics regardless of sex, emphasizing the importance of considering recovery timing when scheduling COD-related assessments or training tasks following maximal anaerobic efforts.

### Limitations

Several limitations of the present study should be acknowledged. First, the assessment of change-of-direction performance was limited to a single planned COD test (Illinois test), which does not include reactive or perceptual–cognitive components of agility; therefore, the findings cannot be generalized to reactive agility performance. Second, neuromuscular, biomechanical, and physiological variables (e.g., electromyography, kinematic analysis, blood lactate, or muscle oxygenation) were not directly measured, limiting the ability to confirm the underlying mechanisms responsible for the observed performance changes. Third, COD performance was evaluated only during the early recovery period (up to 10 min post-exercise), and longer recovery durations were not examined. Finally, although the sample included both female and male participants, the findings may not be generalizable to elite athletes or sport-specific populations with different training backgrounds. Future studies incorporating reactive COD tasks, direct neuromuscular measurements, and extended recovery monitoring across different athletic populations are warranted.

In addition, although sex was included as a between-subject factor, the present study was not specifically designed to model sex-specific recovery trajectories or to fully explain the observed Group × Sex interaction. The absence of time-dependent interaction effects suggests similar recovery patterns between female and male participants; however, these findings should be interpreted with caution in terms of generalizability. Future studies with larger, sport-specific samples and designs explicitly powered to examine sex-specific recovery responses are warranted to further clarify potential differences in fatigue and recovery dynamics following maximal anaerobic exercise.

## Figures and Tables

**Table 1:** Descriptive Statistics of Height, Weight, and BMI Values of Athletes Participating in the Study.

Parameters	Min	Max	Mean	SD
Height	157.00	193.0	172.9	9.48
Body Weight	45.00	103.0	66.49	12.55
BMI	16.94	29.07	22.09	2.74

**Table 2:** Descriptive statistics for performance values across five time points.

Time	Group	Female (Mean ± SD)	Male (Mean ± SD)
Baseline	Experiment	17.8960 ± 0.71096	16.4905 ± 1.15321
Baseline	Control	17.2012 ± 0.72573	16.8092 ± 1.50931
4 min	Experiment	18.5467 ± 0.96238	17.1545 ± 1.72184
4 min	Control	16.9488 ± 0.73684	16.7715 ± 1.35017
6 min	Experiment	19.0813 ± 0.98058	17.0413 ± 1.26310
6 min	Control	17.1665 ± 0.64017	17.0785 ± 1.65841
8 min	Experiment	19.0193 ± 1.20136	16.7680 ± 1.21136
8 min	Control	17.4141 ± 0.77982	17.4585 ± 1.65465
10 min	Experiment	19.0487 ± 1.65877	17.2830 ± 1.54905
10 min	Control	17.2782 ± 0.68244	18.1854 ± 1.11969

**Table 3:** Mixed ANOVA results examining the main and interaction effects of Time, Group, and Sex on performance outcomes.

Effect	F(df)	*p*-Value	Partial η^2^
Time	6.48 (4.58)	<0.001	0.309
Time × Group	2.04 (4.58)	0.099	0.123
Time × Sex	1.18 (4.58)	0.324	0.075
Time × Group × Sex	1.86 (4.58)	0.128	0.114
Group	7.15 (1.61)	0.010	0.105
Sex	14.47 (1.61)	<0.001	0.192
Group × Sex	16.53 (1.61)	<0.001	0.213

## Data Availability

The raw data supporting the conclusions of this article will be made available by the authors on request.
